# SOCIAL RELATIONSHIP TRAJECTORIES AND HEALTH OUTCOMES IN LATER LIFE

**DOI:** 10.1093/geroni/igad104.2196

**Published:** 2023-12-21

**Authors:** Yingzhi Xu

**Affiliations:** Duke University, Durham, North Carolina, United States

## Abstract

Many studies have demonstrated the strong causal association between social relationships and health. Research acknowledges social relationships as a “double-edged” phenomenon—social support favors longevity, while social strain fosters disadvantages in health. However, little research has investigated how social support and social strain develop over the life course. The growth mixture model (GMM) and four waves of the Health and Retirement Study (HRS) were used to identify group-based trajectories of perceived social support and social strain from four categories of social connections: spouse, children, family, and friends. Ordinal logistic regression was employed to examine the degree of exposure to social support and social strain and their effects on physical and psychological health among older populations in the U.S. Four classes of support trajectories involving children, family, and friends, three classes of spousal support trajectories, four classes of strain trajectories involving spouses, children, and friends, and two classes of friend strain trajectories were identified. The majority of older adults have experienced stable high social support and low social strain from four categories of social connections. As an emotionally closer tie, the perceived social support and social strain from spouses are highest among the four categories. Friends provide the second highest social support, while they are the lowest in terms of social strain as a weaker tie. People perceived stable high social support, and low social strain have better psychological health compared to other groups. The findings revealed the social support and strain patterns of different social connections and related health consequences.

